# Combining Phi6 as a surrogate virus and computational large‐eddy simulations to study airborne transmission of SARS‐CoV‐2 in a restaurant

**DOI:** 10.1111/ina.13165

**Published:** 2022-11-27

**Authors:** Lotta Oksanen, Mikko Auvinen, Joel Kuula, Rasmus Malmgren, Martin Romantschuk, Antti Hyvärinen, Sirpa Laitinen, Leena Maunula, Enni Sanmark, Ahmed Geneid, Svetlana Sofieva, Julija Salokas, Helin Veskiväli, Tarja Sironen, Tiia Grönholm, Antti Hellsten, Nina Atanasova

**Affiliations:** ^1^ Department of Otorhinolaryngology and Phoniatrics – Head and Neck Surgery Helsinki University Hospital Helsinki Finland; ^2^ Faculty of Medicine University of Helsinki Helsinki Finland; ^3^ Finnish Meteorological Institute Helsinki Finland; ^4^ Faculty of Biological and Environmental Sciences University of Helsinki Helsinki Finland; ^5^ Faculty of Biological and Environmental Sciences University of Helsinki Lahti Finland; ^6^ Finnish Institute of Occupational Health Helsinki Finland; ^7^ Faculty of Veterinary Medicine, Food Hygiene and Environmental Health University of Helsinki Helsinki Finland; ^8^ Department of Virology, Faculty of Medicine University of Helsinki Helsinki Finland; ^9^ Department of Veterinary Biosciences, Faculty of Veterinary Medicine University of Helsinki Helsinki Finland

**Keywords:** aerosol transmission, air purifiers, COVID‐19, infection‐probability, infective viruses, space dividers

## Abstract

COVID‐19 has highlighted the need for indoor risk‐reduction strategies. Our aim is to provide information about the virus dispersion and attempts to reduce the infection risk. Indoor transmission was studied simulating a dining situation in a restaurant. Aerosolized Phi6 viruses were detected with several methods. The aerosol dispersion was modeled by using the Large‐Eddy Simulation (LES) technique. Three risk‐reduction strategies were studied: (1) augmenting ventilation with air purifiers, (2) spatial partitioning with dividers, and (3) combination of 1 and 2. In all simulations infectious viruses were detected throughout the space proving the existence long‐distance aerosol transmission indoors. Experimental cumulative virus numbers and LES dispersion results were qualitatively similar. The LES results were further utilized to derive the evolution of infection probability. Air purifiers augmenting the effective ventilation rate by 65% reduced the spatially averaged infection probability by 30%–32%. This relative reduction manifests with approximately 15 min lag as aerosol dispersion only gradually reaches the purifier units. Both viral findings and LES results confirm that spatial partitioning has a negligible effect on the mean infection‐probability indoors, but may affect the local levels adversely. Exploitation of high‐resolution LES jointly with microbiological measurements enables an informative interpretation of the experimental results and facilitates a more complete risk assessment.

**Practical implications**

- Novel combination of microbiological measurements and turbulence‐resolving flow modeling indoors is demonstrated.
- Bacteriophage Phi6 functions well as a safe model for SARS‐CoV‐2 and other coronavirus dispersion modeling in indoor environments.
- The enduring infectivity of airborne viruses substantiates the relevance and significance of measures to reduce the risk of long‐range airborne transmission indoors.
- Space partitioning cannot be considered as a viable risk‐reduction strategy against airborne transmission.
- Air purifiers, which augment the existing ventilation capacity and enhance the mixing of indoor air, can act as an effective means to reduce transmission risks indoors.

## 1 INTRODUCTION

Severe acute respiratory syndrome coronavirus 2 (SARS‐CoV‐2)^1^ has caused the most severe pandemic of contemporary history. Despite high vaccination rates of the population, lifting of infection control restrictions has led to a clear increase in infection rates and deaths in several countries forcing them to reannounce infection mitigation measures.^2^ Non‐pharmaceutical interventions and layered mitigations are still needed to reduce infection rates and enable safe social interaction, However, there are still questions about the effectiveness of various safety measures. One key element to tackle is asymptomatic spreading, where the virus is shed most actively before the first symptoms or during the initial, often still mild, symptoms allowing the host to attend events and gatherings.^3^ This leads to a question; how can we prevent indoor transmission and reduce the risk associated with social meetings during the pandemic?

The risk of infection transmission has been recognized to be higher indoors than outdoors, causing most of the secondary cases.^4^, ^5^ Correspondingly, SARS‐CoV‐2 RNA has been detected at higher concentrations indoors than outdoors.^6^ As can be expected, the highest positivity rates from air samples have been close to a known source of infection.^6^ Additionally, SARS‐CoV‐2 has been detected in multiple air samples, in multiple particle sizes with an emphasis on small aerosols.^7^, ^8^, ^9^, ^10^, ^11^, ^12^ After a wide scientific discussion during the pandemic, there is a strengthening consensus that SARS‐CoV‐2 spreads mainly by both short and long‐distance airborne transmission via infective aerosol particles.^13^, ^14^, ^15^, ^16^, ^17^, ^18^, ^19^ Supporting this, several transmission events have been observed without any close contact between individuals.^20^, ^21^, ^22^, ^23^ Particles containing potentially infectious viruses are produced during normal respiration, speaking, singing and coughing and at higher emission rates as the amplitude rises.^24^, ^25^, ^26^ The role of small aerosol particles, less than <5 μm in size, as potential sources of disease is important, as over 99% of the produced particles fall into this size class.^25^ This study focuses on long‐distance aerosol transmission, omitting the immediate vicinity of infection source (distances less than roughly 2 m) where direct droplet transmission may occur and where aerosol concentrations are greatest and highly specific to the nature of the respiratory activity (e.g., coughing, sneezing, or laughing).

We study airborne transmission and potential fomite transmission following aerosol particle deposition on surfaces in one of the environments that has been restricted globally: a restaurant. Restaurant settings are essentially environments where people sit in rather small rooms for a prolonged time, often without using masks or respirators so as to allow eating and drinking. When an infected individual is present, such spaces see an increase in virus‐laden airborne particle concentrations as the viral host continues to breath, speak, laugh and possibly cough. Environmental factors such as temperature, humidity, and ambient airflow significantly affect the transmission and dispersion of aerosol particles that may carry microbial agents^18^; the use of an actual restaurant environment offers a unique opportunity to address the complexity of respiratory aerosol dispersion and the ability of airborne viruses to maintain their infectivity indoors. As it would not be ethical to study transmission of the actual SARS‐CoV‐2 virus in a restaurant, we chose to use an enveloped bacteriophage Phi6, a widely used SARS‐CoV‐2 surrogate.

In this study we conducted a novel series of virus‐laden aerosol dispersion experiments in a real restaurant placing emphasis on measurements detecting infectious viruses via airborne transmission. This work documents an approach where experimental outcomes are examined together with time‐ and space‐resolved dispersion results obtained from high‐resolution large‐eddy simulation (LES) modeling, which replicates the experimental setup. Although the LES modeling cannot provide measures directly comparable to the experimental samples, for example, due to the temporally cumulative nature of microbiological measurements, the model results provide complementary evidence on the dispersion problem, which is critical in the interpretation of infectious virus results. The LES model has been validated against experimental aerosol dispersion measurements in our previously published article.^27^ This study seeks to uncover evidence, which will improve our understanding concerning the airborne transmission mode of respiratory pathogens and answer the following research questions:

1. Do airborne viruses maintain their infectivity indoors despite prolonged residence times and traversed distances?
2. What is the significance of surface contamination resulting from the deposition of airborne pathogens?
3. How do risk‐reduction strategies influence the evolution of spatial infection probability?

The studied risk‐reduction strategies included (a) the increase of effective ventilation and mixing of the air using air purifiers (FLT), (b) use of space dividers (DIV) and (c) combining space dividers with air purifiers (DIV + FLT).

## 2 MATERIALS AND METHODS

### 2.1 Summary of the simulation protocol

**FIGURE 1:**
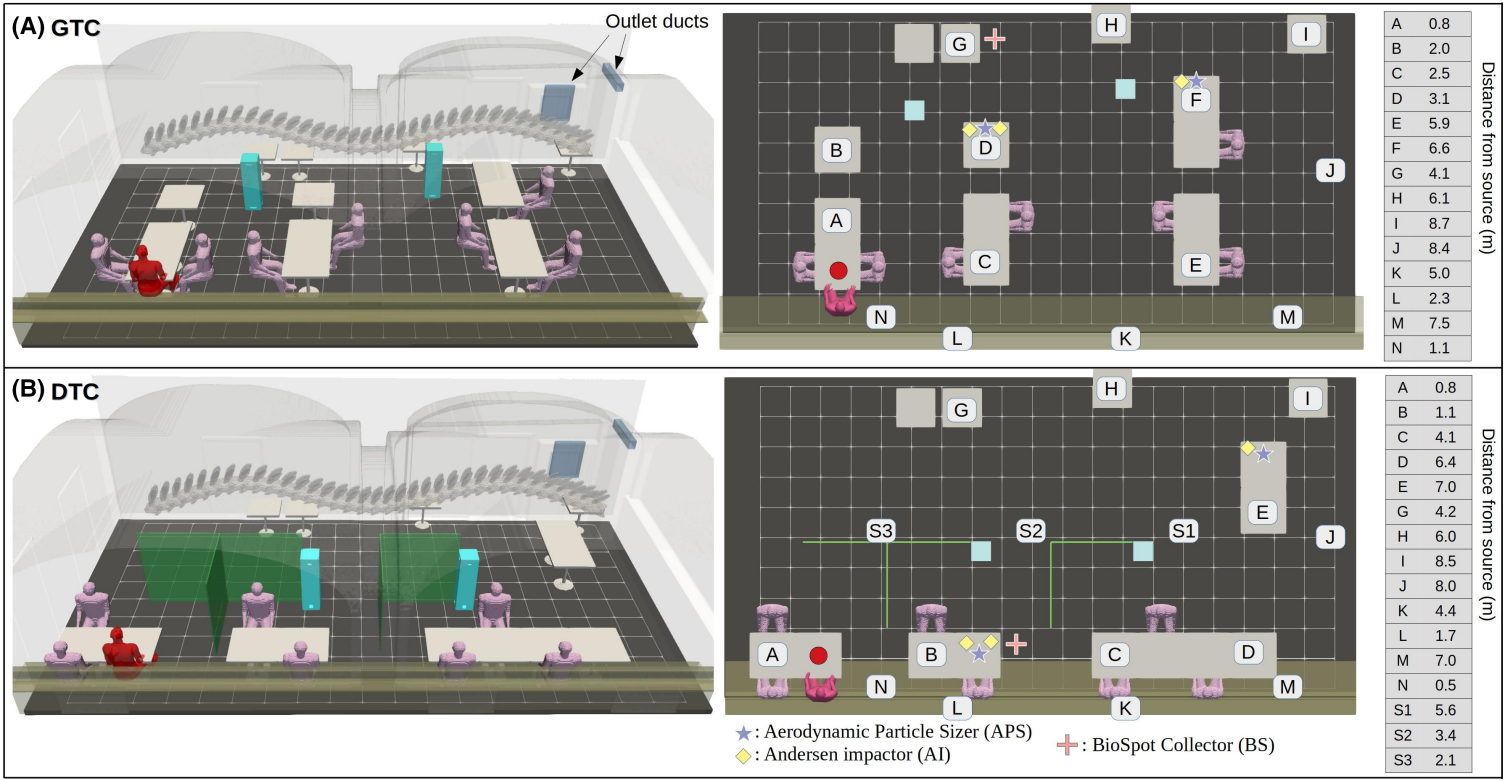
A three‐dimensional visualization of the experimental layout at the restaurant for (A) the Generic Table Configuration (GTC) and (B) for the space‐Divider Table Configuration (DTC). The top view on the right illustrates the relevant sensor placements together with their tabulated distances from the nebulizer unit, which is colocated with the imaginary infected individual depicted in red color. The three‐dimensional model is also used to construct a large‐eddy simulation (LES) model, which replicates the ventilation, thermal and structural complexity of the real space.

**FIGURE 2:**
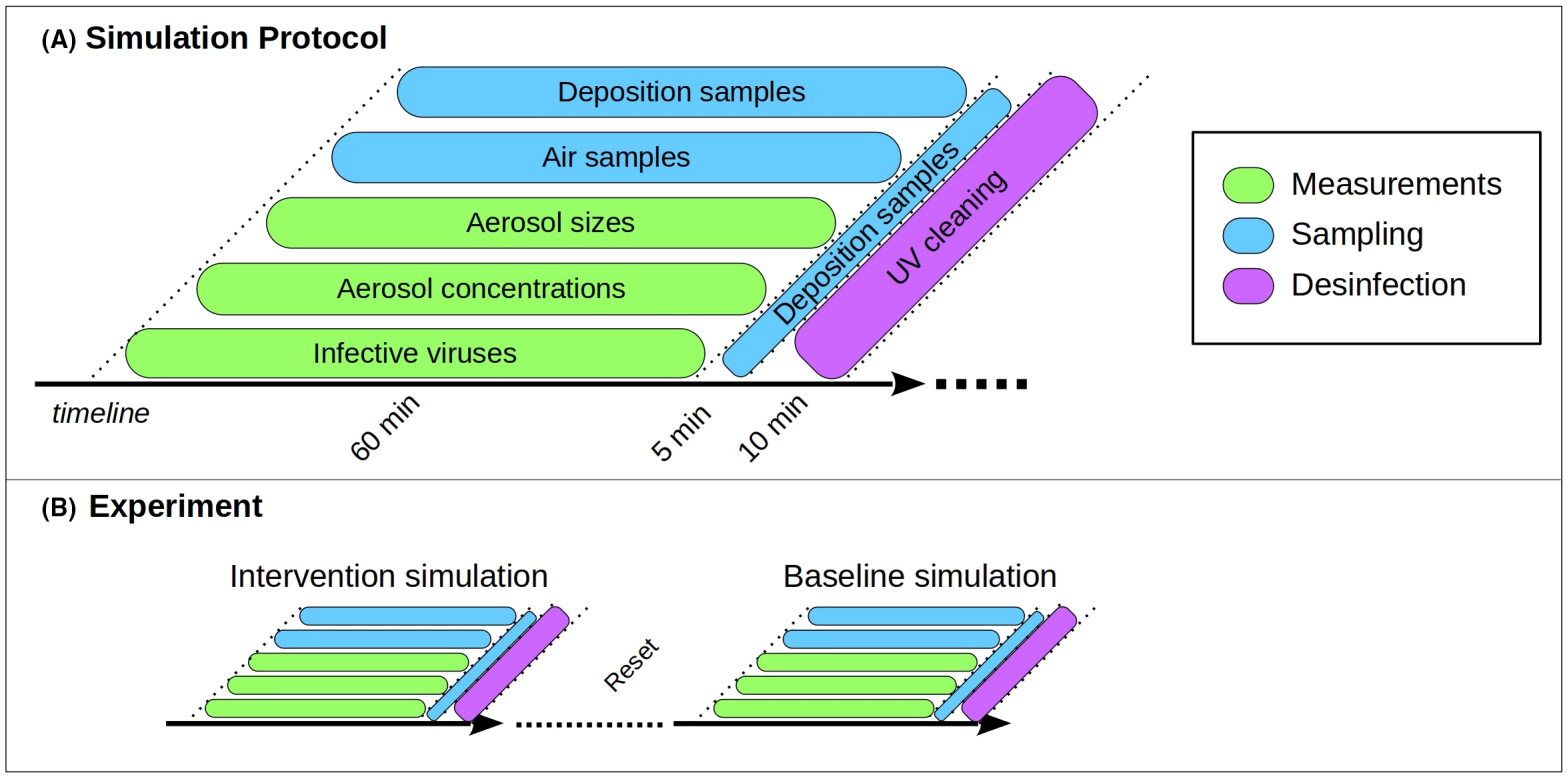
A decomposition of the simulation protocol (A) and a schematic of the experimental plan for each simulation day (B).

All simulations were conducted in the Restaurant Ultima in Helsinki, Finland between November 16th, 2020 and February 1st, 2021. A single dining area, approximately 60 m^2^ (170 m^3^) was selected for the experiment and isolated from other restaurant rooms, see Figure 1. The restaurant has mechanical ventilation with a baseline ventilation rate in this test configuration 820 m^3^h^−1^ corresponding to 4.8 air changes per hour.^27^ The simulation protocol and the experiment plan are laid out in Figure 2.

To assess infection probabilities, it is crucial to understand the dispersion of the aerosol particles in the space as accurately as possible. To achieve this, the aerosol spread and indoor turbulence in the dining room was studied with an LES model validated for this space in our previous study^27^ and with APS‐measurements. Preparatory 90 min + 30 min simulation was performed to assess temporal scales of the dispersion (aerosol concentration increase and decrease) and ensure the functionality of the study protocol. Subsequently, various risk‐reduction interventions were studied with two consecutive 60 min experiments per day: a reference simulation and an intervention simulation including the studied risk‐reduction strategy (use of air purifiers or space dividers or both), see Figure 2. It should be noted that each intervention run had its own reference run performed on the same day to ensure similar environmental conditions, such as relative humidity and temperature, that may affect aerosol particle size and retention of infectivity.^28^ The effects of varying distances from the virus source were assessed in all simulations, see Table A1 for sampling points.

Two different table configurations were employed, see Figure 1: a baseline configuration labeled generic table configuration (GTC) and a modified table configuration to enable the use of space dividers, labeled divider table configuration (DTC). Overall, three different risk‐reduction strategies with the suggested potential to reduce pathogen exposure indoors were studied as well as the exposure time and distance aspects. These strategies were:

1. enhanced ventilation capacity with air purifiers (APs), abbreviated as GTC:FLT with the applicable table configuration;
2. altered spatial partitioning with space dividers, abbreviated as DTC:DIV;
3. combining 1 and 2, abbreviated DTC:DIV + FLT.

#### 2.1.1 Aerosol concentration increase and decrease

Before studying the interventions, the temporal scales of the concentration increase and decrease were studied both experimentally and numerically. An experimental simulation of a 90 min aerosolization period and subsequent 30 min removal period was first carried out in order to ensure that the selected study period of 60 min was sufficient for aerosol particles to spread everywhere in the room and to investigate their flushing after nebulization was stopped. This 90 min + 30 min simulation was made in the GTC‐configuration with no interventions. Deposition samples were collected on two adjacent petri dishes containing host bacterium. The lid of one dish was left open, while the other's lid was closed during the 90 min nebulization period. Afterwards, the open lids were closed and closed lids were opened for the last 30 min with the nebulization off.

#### 2.1.2 Risk‐reduction concepts

The first risk‐reduction concept, GTC:FLT, used enhanced ventilation by introducing two portable UniqAir PRO^29^ air purifiers, augmenting the existing baseline ventilation rate by 540 m^3^h^−1^, representing an increase of 65%. The air purifiers employed both HEPA and active carbon filters in series with a reported 99.97% or higher filtration rate for particles, which are larger than 0.1 μm in diameter.^29^ The placement of the air purifiers is shown in Figure 1. The second strategy, DTC:DIV, utilized spatial partitioning where the dining tables were separated from each other using 1.5 m tall plexiglass panels, see Figure 1. The third strategy DTC:DIV + FLT combined the use of both partitioning and air purifiers simultaneously.

#### 2.1.3 Summary of sample collection

Air samples (see Section 2.3.1) were collected using both active and passive sampling during the simulations, while surface samples (see Section 2.3.3) were collected immediately after the nebulization ended. All samples were immediately placed on ice and transported to a microbiological laboratory. The transportation time was approximately 40 min. Aerosol particle concentration and size distribution (see Section 2.3.2) were analyzed throughout the simulations. Following each simulation, the test room was disinfected with ultra‐violet (UV) radiation after sample collection (see Appendix 1).

#### 2.1.4 Human involvement and ethical considerations

Seven researchers stayed in the dining room throughout each simulation. The placement of the measurement devices, analysis tools and the seven people involved in the simulations can be seen in Figure 1. All participants were given personal protective equipment including FFP2/3 respirators, eye protection, hair protection, fabric coveralls, gloves and shoe protection. All procedures were conducted in accordance with the ethical standards of the 1964 Declaration of Helsinki and its later amendments. The Ethics Committee of Helsinki University Hospital approved the study protocol (HUS/1701/2020) and all persons involved in the simulation gave informed consent prior their participation.

### 2.2 Virus strain, preparation and nebulization

#### 2.2.1 Phi6 as a surrogate

The enveloped bacteriophage Phi6 (family *Cystoviridae* that infects *Pseudomonas syringae* bacteria) was used as a surrogate for SARS‐CoV‐2. Phi6 is widely used to study coronaviruses including SARS‐CoV‐1 and SARS‐CoV‐2^30^, ^31^, ^32^ as it resembles the SARS‐CoV‐2 virus in size ~80–100 nm and surface structure, as well as in retention of infectivity. It is also shown to be suitable for viral aerosol studies^33^ because it is harmless to humans, plants, and animals. Due to these properties, Phi6 is the most suitable as a safe mechanical model for assessing SARS‐CoV‐2 virus transport under real, indoor ventilation conditions.

#### 2.2.2 Virus strain, preparation and analysis of infectious virus

Phi6 viruses were produced and purified as described according to Bamford et al.^34^ and *Pseudomonas syringae* pathovar phaseolicola (HB10Y) strain was used as the virus host. The Phi6 was originally kindly provided to the research group by Dr Anne K. Vidaver.^35^ Purified Phi6 viruses were diluted in a 20 mmol L^−1^ potassium‐phosphate (K_3_PO_4_) in 1 mmol L^−1^ MgCl_2_ (pH 7.2) buffer with a final infectious‐particle concentration of approximately 10^10^ pfuml^−1^. The solution was kept on ice during transfer to the restaurant from the laboratory. The virus‐laden aerosol particles were generated using an Omron Ultrasonic Nebulizer Model NE‐U17 to mimic the exhalations of an infected human. A detailed description of the nebulization arrangement is found in Ref. [27].

The presence of infectious virus particles in air and deposition samples was demonstrated by plating on the virus host, HB10Y, which was grown in Luria‐Bertani medium at 22°C with aeration. Culture petri dishes were prepared by mixing 200 μl of exponentially growing HB10Y and 3 ml of Luria soft agar on a solid Luria agar plate. Plaque assay was used to measure the titer of infectious Phi6 particles in the samples after overnight incubation at 22°C. Individual plaque‐forming unit (pfu) can be detected and counted with sufficient accuracy up to 700 units per plate while higher values cannot be specified and are hereby marked as >700.

#### 2.2.3 Nebulization

We filled the nebulizer with 150 ml of virus solution at the beginning of each simulation. The nebulization rate was approximately 0.3 ml min^−1^ and the air volume flow rate was approximately 141 min^−1^. The nebulizer produced 6–8 μm sized wet particles and the mode of the dry size distribution was approximately 0.9 μm while relative humidity was approximately 28%.^27^

During nebulization, the liquid is warmed to 33–35°C during 30 min of use. Because the Phi6 virus is sensitive to temperature increases and not able to stay viable above 25°C,^31^, ^36^ we had to cool the system. The virus solution was kept below 5°C temperature until inserted into the liquid chamber of the nebulizer, which was covered by a cooling element. After 30 min, the virus solution was replaced with a fresh, cooled sample. With this setup, the aerosol temperature measured at the outlet was 8–12°C after 5 min and 16–20°C at the end of the 30 min cycle. The viability of the virus and the virus concentration were verified after nebulization from each used solution to assure the comparability of the samples (Table A2 in Appendix 2). In order to ensure the constant rate of aerosolization of infectious viruses during nebulization, assessment deposition plates containing the host bacterium were placed 0.8 m from the nebulizer, see Figure 1, and cumulative virus deposition was measured for every 30 min period in all 60 min and 90 min simulations (Table A3 in Appendix 2).

Due to the evaporative cooling taking place after the aerosol exits the nebulizer outlet, the plume becomes negatively buoyant and tends to sink. The sinking is an undesired phenomenon since the aim is to mimic human exhaling, which is positively buoyant. To alleviate this discrepancy, a thin heated stainless steel plate (dimensions 44 ×59 cm) with a 20 W heating cable installed under the plate was placed below the outlet. The aerosol temperature above the plate surface was measured and found to vary between 16°C and 19°C while the ambient air temperature of the room was roughly 19°C. Hence, the combined thermal and mechanical effects reduced the sinking significantly.

### 2.3 Measurements

#### 2.3.1 Air sample collection

Active air samples were collected using the BioSpot 300p (Aerosol Devices Inc.) bio‐aerosol sampler prototype with 81 min^−1^ flow rate and two Andersen cascade impactors with 28.31 min^−1^ flow rate. The BioSpot collects infective viruses by condensing 5 nm–20 μm aerosol particles to water thereby minimizing the mechanical stress during collection. The BioSpot is supplied with a system meant for securing the gentle transfer of the sample with eight wicking tubes fitted with three nozzle jets. The samples were collected to 1 ml of HEPES with 60 min collections. The Andersen impactors consist of six size distribution stages of decreasing hole sizes toward the base, and were fitted with metal inlets of 12 μm cut point (EPA designed). The size ranges of the collection stages were (1) 7–12 μm, (2) 4.7–7.0 μm, (3) 3.3–4.7 μm, (4) 2.1–3.3 μm, (5) 1.1–2.1 μm, and (6) 0.65–1.1 μm. The flow rate of each Andersen impactor was controlled with a flow regulator and TSI flow meter. Andersen impactors 1 (near) and 2 (far) were each filled with six Luria‐HB10Y culture petri dishes and were used to measure the concentration of infective viruses in the air. Initially the Andersen collectors were used for 30 min in GTC:FLT simulation. However, as the pfu results were often larger than 700 too many to count (TMC), the collection time was reduced to 20 min for the DTC:DIV simulation and further to 10 min for the DTC:DIV + FLT simulation. Thus, the Anderson results are valid within each simulation and its reference separately, but direct comparisons between different simulations cannot be done.

Passive air samples were collected by deposition directly onto petri dishes containing Luria‐HB10Y agar. Plates were distributed throughout the room according to the layout shown in Figure 1. Deposition samples were collected for 60 min during nebulization.

#### 2.3.2 Aerosol particle measurements

Real‐time aerosol particle concentrations were measured using two model 3321 Aerodynamic Particle Sizers (APS; TSI Inc.). APS is a time‐of‐flight‐based aerosol spectrometer, which measures the aerodynamic size of particles from 0.5 to 20 μm with a 52‐bin resolution. The time resolution of the measurements was 10 s. The APS's were equipped with total suspended particle (TSP) inlets, and the sampling was conducted from a height of 1.25 m.

#### 2.3.3 Surface sample collection

Samples were collected from surfaces by two methods: (1) Swab samples were taken by introducing a sterile polyester swab in HEPES buffer, swabbing an approximately 10 × 10 cm surface next to the deposit sample plates seen in Figure 1 and scissoring the swab head into 1 ml of HEPES buffer. Plaque assay was used to measure the titer of infectious viruses. (2) With a sterile glove, table surfaces were touched by two fingers, which were subsequently inoculated on HB10Y culture petri dishes, incubated overnight at 22°C and visually examined for cell lysis by Phi6 virus (positive or negative at the place of finger).

### 2.4 Dispersion modeling

The computational dispersion modeling of aerosol dispersion indoors was performed with PALM LES model.^37^, ^38^ A version of PALM was specifically modified and adapted for the present indoor flow problem by Auvinen et al.^27^ The indoor ventilation flow model was constructed from a detailed 3D description of the room used in the experiments. The infected individual in the model was implemented in a fully scalable manner as a unit concentration source and its location (shown in red in Figure 1) was coincident with the nebulizer device used in the experiments (see Section 2.2). The flow and thermal boundary conditions of the numerical model were specified in accordance with the conditions observed during the experiments. The LES simulations describing the dispersion of virus‐laden aerosol particles were run for 60 min to imitate the experimental protocol laid out in Figure 2. The modeling neglects deposition onto surfaces as this mechanism is deemed, based on the deposition results, unable to alter the concentration in the room within detectable limits. The removal simulations, accounting for the period after the infected individual has left the room, were carried out for 45 min. A detailed description of the numerical models, their validation and application to infection‐probability analysis are documented in Ref. [27].

Different LES model variations were constructed to correspond with each spatial and ventilation configuration considered herein. Their 3D structural representations, which are shown in Figure 1, were used in visualizing both numerical and experimental results wherever convenient.

### 2.5 Estimation of infection probability

Effects of the interventions on infection probabilities were estimated based on the LES‐predicted concentration data. Infectious pathogens must reach the target receptors and survive the immune defense; thus in most cases more than one virion is needed to cause an infection. The term *quantum* describes the infectious dose that is needed to develop a disease.^39^ The exact number of viruses needed to achieve a quantum for SARS‐CoV‐2 is unknown, but is likely to be highly variable depending on the variant and resistance properties (i.e., previous infection, immune status, vaccination, region of the original infection) of the host.^40^ We assume here the quanta rate of 100 qh^−1^ from the infected person (nebulizer) and an average breathing rate of 800 dm^3^h^−1^, in accordance with Auvinen et al.^27^ This choice is justified by the log‐normal probability density function (PDF) for the quanta rate proposed by Buonanno et al.^41^ This PDF has its peak value at 20 qh^−1^ and the PDF is larger or equal to 80% of its peak value between 10 qh^−1^ and 100 qh^−1^. We chose to adopt the higher end 100 qh^−1^ of this range. It should be noted that the emitted quanta rate is highly variable and hence there is no single correct value to be chosen. Moreover, the infection‐probability results are always easily scalable for any other quanta‐emission rate.

Spatially variable probability fields are estimated by inserting LES‐predicted time‐ and space‐dependent aerosol concentration fields in an extended version of the Wells‐Riley probability model. This methodology is described in detail by Auvinen et al.^27^ The probability fields are either vertically averaged ${\left\langle P\right\rangle }_{z}$ over the so‐called *living zone* with *z* ranging from 0.1 to 2 m or spatially averaged in all three‐dimensions. The three‐dimensionally averaged infection probabilities $\left\langle P\right\rangle$ are computed such that the averaging spans horizontally over the entire indoor space and vertically over the living zone, while omitting the near‐source space represented by a vertical, circular cylinder with a radius of 2 m centered around the source. The reason for omitting this area is because we focus on the longer‐distance aerosol transmission, rather than the near‐source space where direct droplet transmission may also take place, the infection risk is obviously elevated, and aerosol concentrations are highly specific to the nature of the respiratory activity.^27^ For the purpose of evaluating the effectiveness of proposed interventions, we employ relative differences defined as

(1) $$\frac{\Delta {\left\langle P\right\rangle }^{\left(i\right)}}{\left\langle P_{R}\right\rangle }=\frac{\left\langle P_{i}\right\rangle -\left\langle P_{R}\right\rangle }{\left\langle P_{R}\right\rangle }$$

where the subscript *R* refers to the GTC (REF) case and *i* to the considered intervention.

## 3 RESULTS

### 3.1 General results on aerosol dispersion in the restaurant

#### 3.1.1 Temporal scales of the dispersion, 90 min + 30 min simulation

In the simulation, infectious viruses were detected until the end of aerosolization at all deposition plates from 0.6 to 8.7 m from the nebulizer, with the highest virus titers measured at 0.6–2.7 m distances. Higher values were detected also on windowsills, both 2.5 and 5.0 m from the nebulizer.

The accumulated numbers of infectious viruses during the 30 min removal period, that is, after the aerosolization was stopped, were clearly lower in all measurement points than the numbers accumulated during the 90 min aerosolization period or the numbers at the virus titer plates used for each 30 min part of the aerosolization period at 1.6 m distance. The result indicates that concentration of infectious viruses drops significantly after aerosol production ceases. The results are presented in Table 1.

**TABLE 1:** Reduction of infective viruses after nebulization.

Distance from nebulizer (m)	Measurement point	Virus titer after 90 min nebulizing (pfu)	Virus titer 30 min after (pfu)
0.6	Table A	>700	ND1
1.6	Table B	317	2
2.5	Table C	424	16
2.7	Chair near table D	621	10
6.2	Table F	68	4
4.2	Table G	135	7
6.4	Table H	38	4
8.6	Table I	84	1
8.7	Back wall shelf J	49	5
5.0	Windowsill K	386	7
2.5	Windowsill L	221	13
8.0	Couch M	103	4
1.6	Couch N	240	8

Virus titer during first, second and third 30 min of nebulizing (pfu)			
Distance from nebulizer (m)	Measurement point	Time fraction (min)	Virus titer (pfu)
1.6	Couch N	1st 30	67
1.6	Couch N	2nd 30	48
1.6	Couch N	3rd 30	78

*Note*: The upper part of the table presents the cumulative virus titers after 90 min aerosolization period and subsequent 30 min removal period. The virus titers are systematically lower after the nebulization stopped. The lower part of the table presents results from the control deposition samples collected from each 30 min period during nebulization. The sensitivity of the nebulization to salt and virus concentration can be seen similarly compared to APS results presented in Figure 4.

Abbreviation: ND, not determined.

**FIGURE 3:**
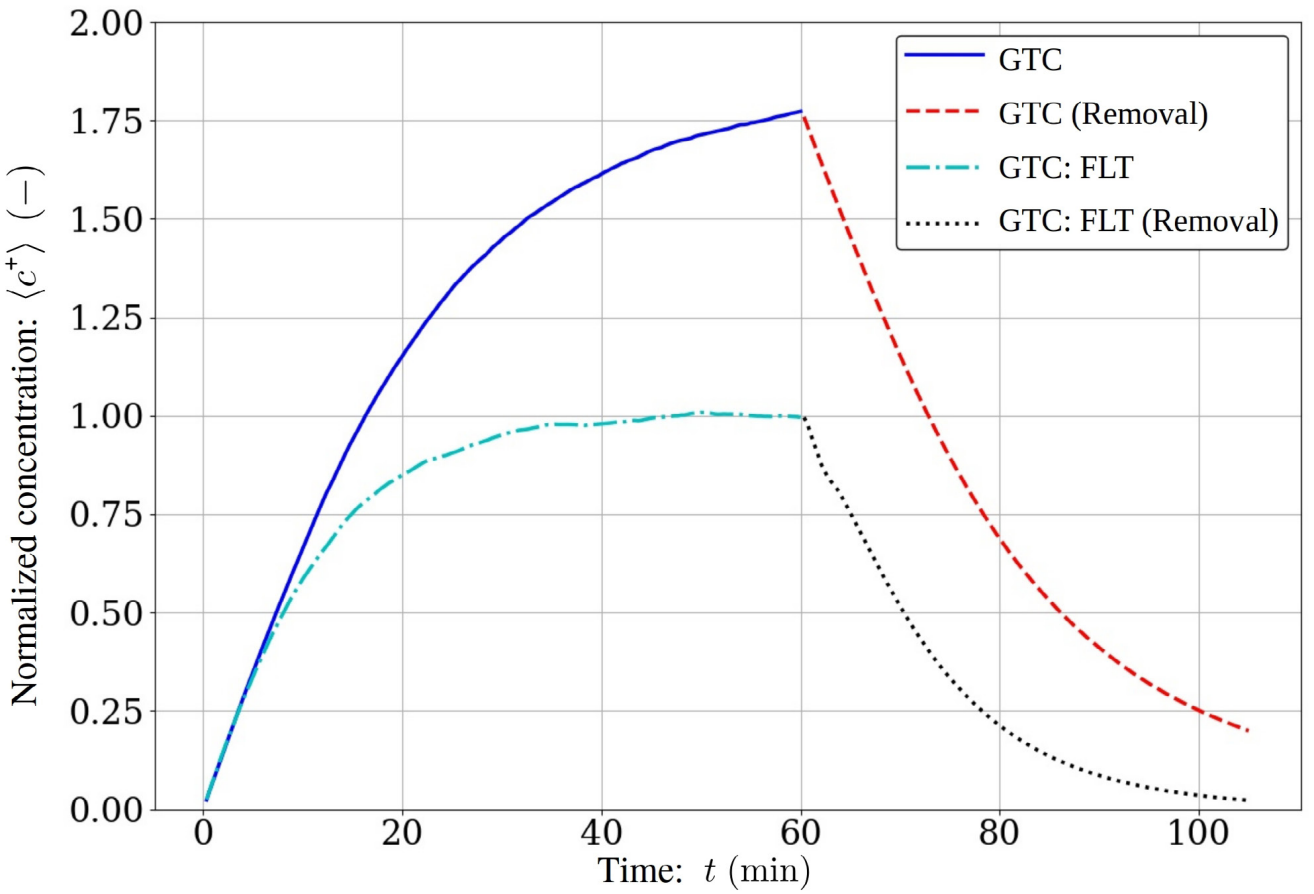
Large‐eddy simulation (LES) modeled evolution of space‐averaged and normalized mean concentration of virus‐laden aerosol particles within the restaurant dining room. The modeling scenario accounts for a 60 min occupation of the infected individual and a subsequent 45 min period after their departure. The concentration is normalized $c^{+}=c/c_{t\to \infty }^{\mathrm{FLT}}$ where $c_{t\to \infty }^{\mathrm{FLT}}$ is the asymptote from the initial GEN + FLT 60 min occupation period.

In order to support the above observations, two LES runs with 60 min aerosolization periods and 45 min removal periods were also performed in the GTC‐configuration, one without any intervention and one with the air purifiers. The time evolution of the normalized mean concentrations $c^{+}=c/c_{t\to \infty }^{\mathrm{FLT}}$ are shown in Figure 3.

The *c* ^+^ decay curves resemble exponential decay quite closely. The concentration half‐lives are approximately 10 and 15 min with and without the air purifiers, respectively. At the end of the removal period, which was 45 min from the end of aerosolization, the concentrations dropped to 2.3% and 11.3% of their previous values at the aerosolization cutoff with and without the air purifiers, respectively.

**FIGURE 4:**
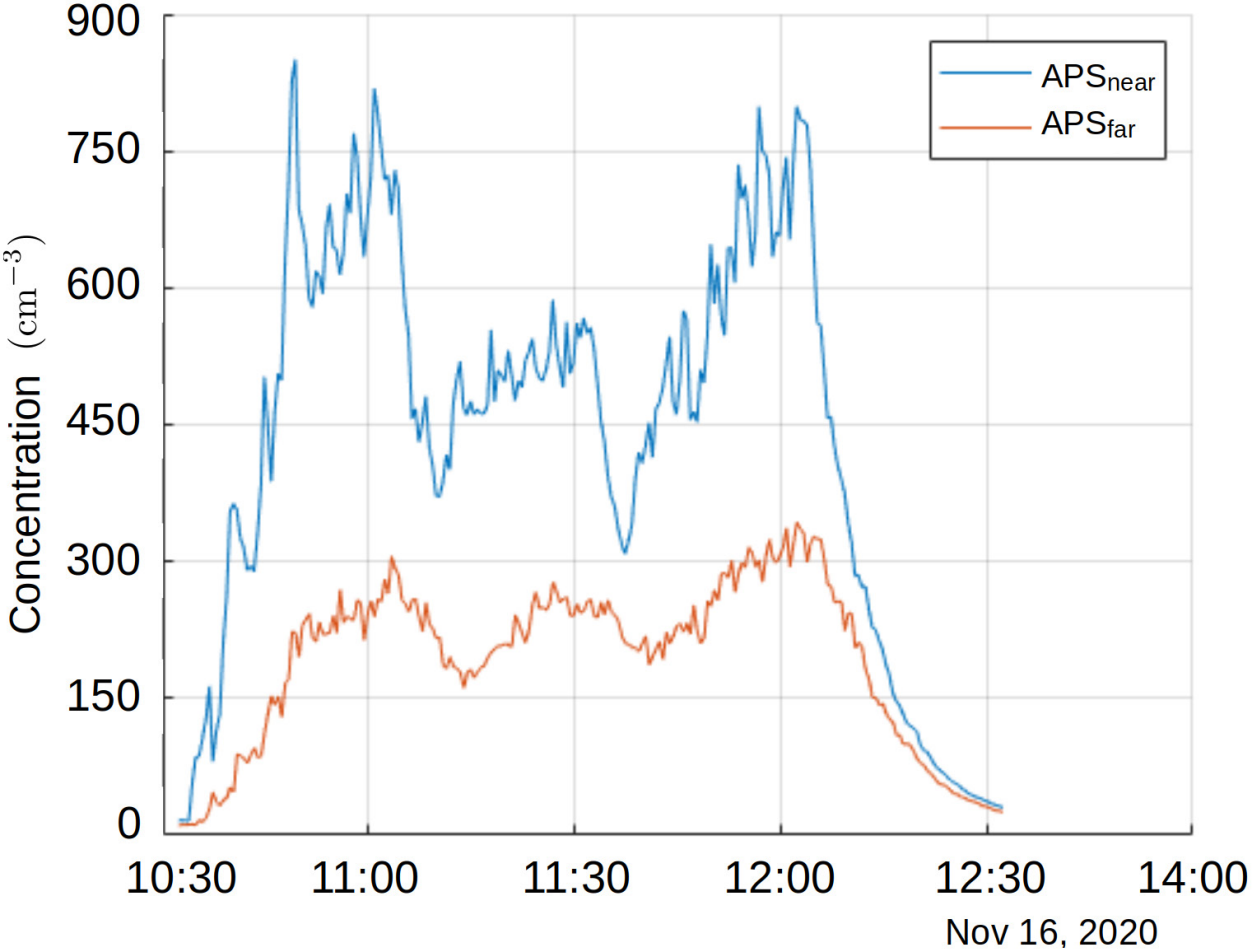
Time series of Aerodynamic Particle Sizers (APS)‐measured particle number concentrations in the 90 min + 30 min simulation (cm^−3^). The position of the APSs in the restaurant is shown in the Figure 1.

The aerosol number concentration time series in the 90 min + 30 min simulation are presented in Figure 4.

#### 3.1.2 Aerosol concentration and size distribution in the studied interventions, 60 min simulations

**FIGURE 5:**
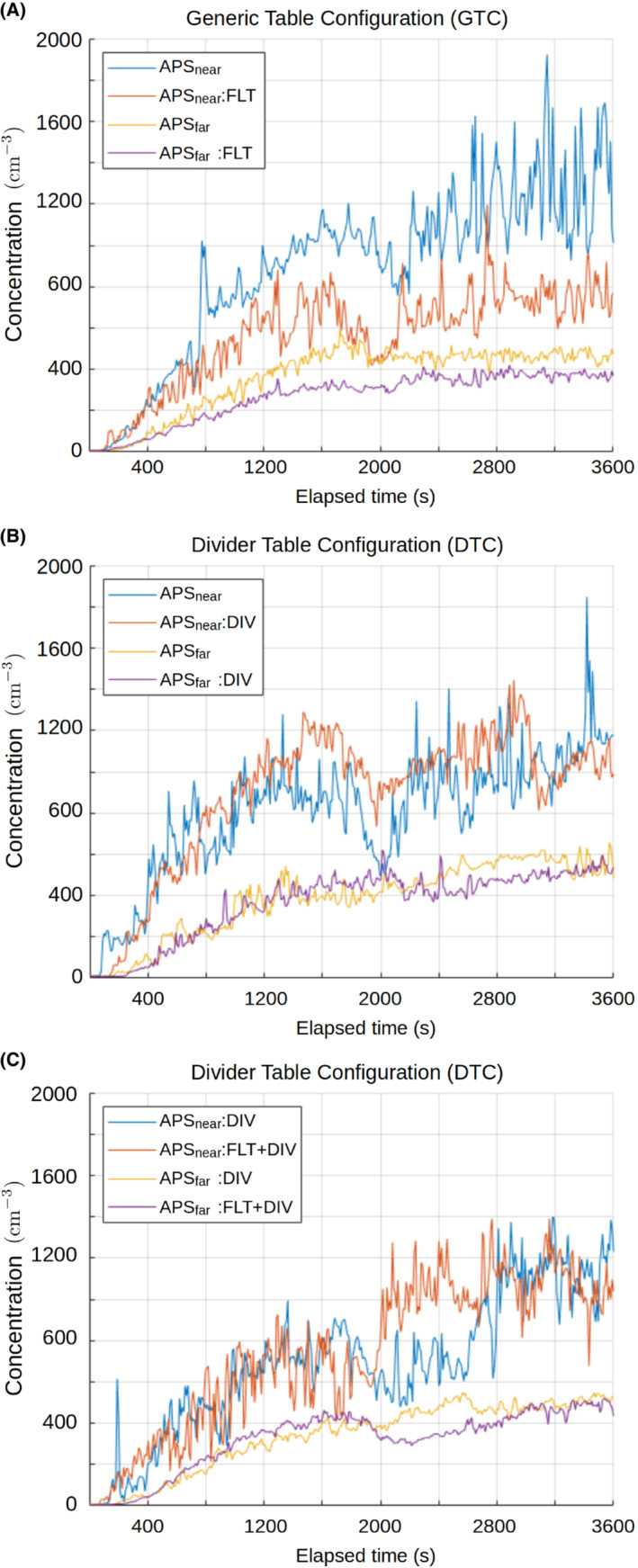
Time series of Aerodynamic Particle Sizers (APS)‐measured particle number concentrations (cm^−3^) in different 60 min simulations.

Plots depicting the evolution of aerosol particle concentrations measured with two APS instruments (APS_near_ and APS_far_) in the studied interventions are shown in Figure 5. The concentration curves exhibit the expected growth pattern, which approaches an asymptotic value. These concentration time series from GTC:FLT and DTC:DIV simulations closely conform with both aerosol dispersion experiments conducted without Phi6 viruses during the LES validation and the LES simulations imitating the experiment.^27^ The highest particle concentrations (1922 cm^−3^) were observed with the APS_near_ closer to the nebulizer in the reference configuration simulation without air purifiers, see Figure 1. Observations made by APS_near_ show higher variability in measured concentrations compared to the APS_far_, which is indicative of incomplete local aerosol mixing.

**FIGURE 6:**
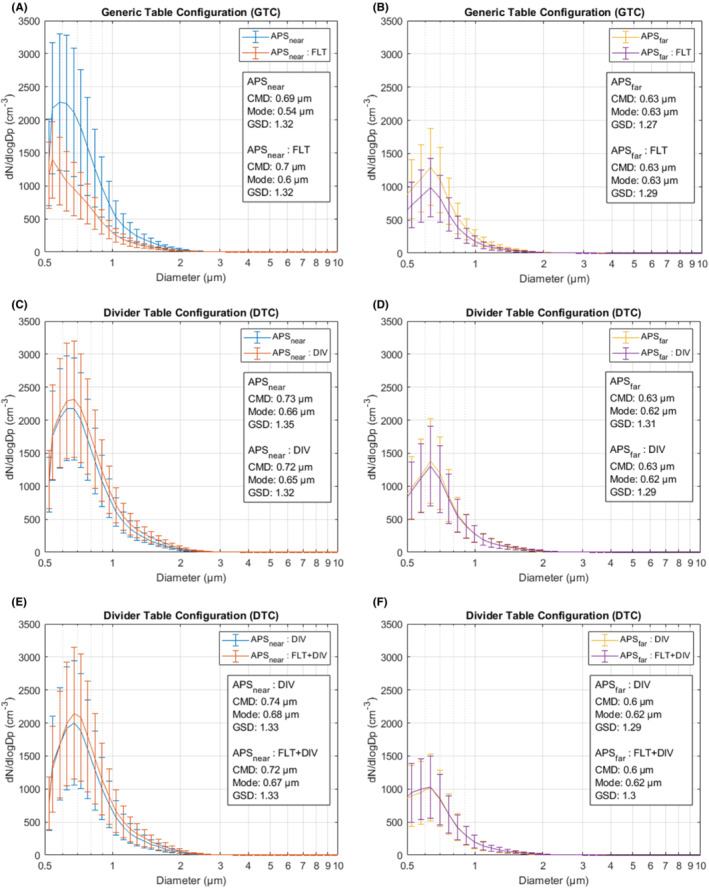
Particle number size distributions and their associated metrics measured with the two APS instruments. The particle count median diameters (CMD) were in the range of 0.60–0.74 μm and the geometric standard deviations (GSD) in the range of 1.27–1.35. The distribution modes varied between 0.54–0.67 μm.

The shapes of the measured particle number size distributions remained similar throughout the different simulations and interventions; the median particle sizes (count median diameter, CMD) are in the range of 0.60–0.74 μm and the geometric standard deviations (GSDs) in the range of 1.27–1.35 (Figure 6). Evaporation equilibrium is reached before the particles reach the APS_near_ as the size distribution remains consistent in both APS measurement points; only the concentrations between APS_near_ and APS_far_ show clear differences.

A supplementary video is prepared to provide an intuitive depiction of pathogen dispersion indoors and demonstrate the subsequent accumulation of infective dose and infection probability within the room. In this context the pathogen source is depicted in terms of quanta rate, which facilitates infection‐probability analysis. The animation can be viewed (or downloaded) https://mega.nz/file/v7pF0aqY#gmFVKcGvXxkw0wfJGCDhBIcQQTP5gXRaqGuj25qaKrk.

### 3.2 Effectiveness of the interventions

#### 3.2.1 Enhanced ventilation capacity (GTC:FLT)

The intervention simulation with the effective ventilation capacity enhanced by two air purifiers (GTC:FLT) shows on average a roughly 30% lower aerosol particle concentration than the reference GTC (REF), see Figure 5. This is true for both APS_near_ and APS_far_, see Figure 1 for exact placement.

**FIGURE 7:**
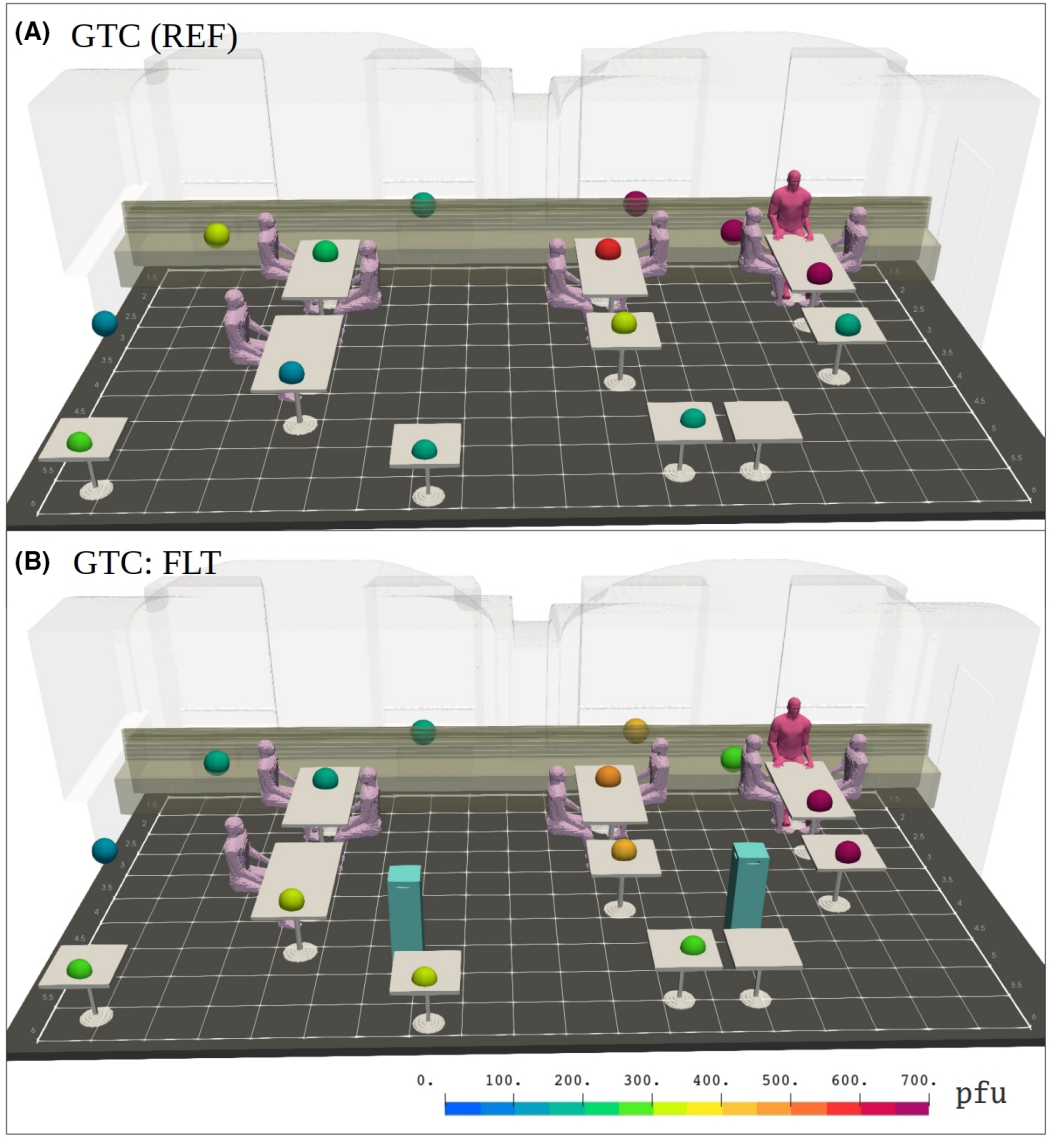
Visualization of plaque‐forming unit (pfu) results on petri dishes from the restaurant simulation employing a generic seating and table configuration (GTC). Normal ventilation conditions are used in (A) whereas ventilation is augmented with two air purifiers in (B). No zero pfu counts were found.

Figure 7 visualizes the deposition‐plate locations with small spheres and the accumulated plaque‐forming unit (pfu) results using color coding for both the reference case GTC (REF) and the intervention case GTC:FLT. The plate locations are also tabulated in Table A1 in Appendix 2. The exact values of infectious viruses per sampling point are shown in Table A4 in Appendix 2.

**FIGURE 8:**
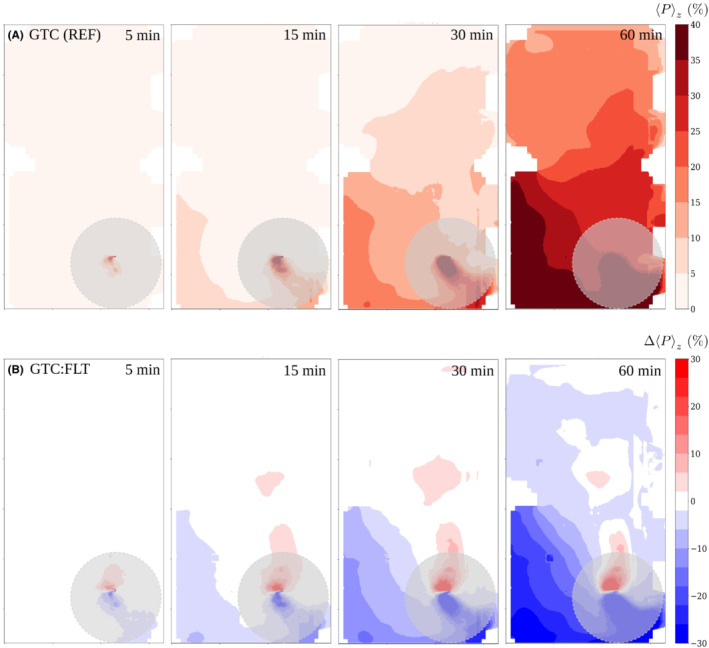
The evolution of infection probability with 100 qh^−1^ quanta rate for the GTC (REF) case (uppermost row), and the absolute difference in percentage units between the air‐purifier intervention GTC:FLT and the GTC (REF) cases. The opaque gray disks having radius of 2 m indicate the near‐source zone within which direct droplet transmission may occur.

Similarly to 90 min + 30 min simulation, infectious viruses were detected in all deposition samples throughout the 60 m^2^ room up to a 10 m distance from the nebulizer. This proves that infectious viruses must transmit in air while maintaining infectivity. Higher aerosol and virus concentrations were seen near the infection source. Compared to the reference (Figure 7A), upon introduction of the air purifiers (Figure 7B), the concentrations are more evenly distributed throughout the room as a result of better mixing of the air. As the mixing, turbulence and airflows near APs are strongly enhanced, also impaction rate to deposition plates increases. Therefore, it must be kept in mind that the deposition‐plate results do not provide a direct, quantitative measure of viral concentrations in the air. The LES‐based infection‐probability results presented later show overall concentration reduction in the same locations (See Figure 8; See Section 4 for details). The same phenomenon was also observed when the APs were combined with the space dividers (DTC:DIV + FLT vs. DTC:DIV) in Section 3.2.3.

Air samples were collected actively with Andersen impactors and the BioSpot sampler, see Table 2. With the APS_far_, lower values of infectious viruses were measured during the FLT simulation in terms of both larger and smaller particle sizes. This result is in line with the LES‐based infection‐probability predictions. Samples collected with the BioSpot do not show significant differences in the concentrations of infectious viruses in either the FLT or REF simulations.

**TABLE 2:** Simulations with air purifiers (GTC:FLT) and the reference (GTC (REF)).

Andersen impactors	GTC (REF)		GTC:FLT	
Particle size *D*_p_ (μm)	A_near_	A_far_	A_near_	A_far_
7 ≤ *D*_p_	112	113	>700	20
4.7 ≤ *D*_p_ < 7	274	317	>700	74
3.3 ≤ *D*_p_ < 4.7	>700	>700	>700	>700
2.1 ≤ *D*_p_ < 3.3	>700	>700	>700	>700
1.1 ≤ *D*_p_ < 2.1	>700	>700	>700	>700
0.65 ≤ *D*_p_ < 1.1	>700	>700	649	475
BioSpot sampler	GTC (REF)		GTC:FLT	
	pfu/ml	pfu/L	pfu/ml	pfu/L
	4.50 × 10^3^	9.40	3.50 × 10^3^	7.30

*Note*: Viable virus counts (pfu) in two different Andersen impactors and the BioSpot sampler. Andersen impactor APS_near_ located 2 m and APS_far_ located 8.0 m from the source. BioSpot sampler collected air samples for the estimation of viable virus concentrations in the air, located at 4.5 m from the source. The unit pfu/ml refers to the amount of infectious viruses in the collection buffer and the unit pfu/L is a derived concentration expressing the amount of infectious viruses in 1 L of air.

All surface swab samples (A–N + Bunny chain, see Figure 1) remained negative when the samples were studied by plaque assay. Finger tests from tables A and E detected infectious viruses in both the reference and intervention simulations indicating the potential for fomite transmission, for example, when touching a contaminated table surface and subsequently one's mucous membranes with the same finger.

The effect of air purifiers on the space‐ and time dependent infection‐probability field is shown in Figure 8 for elapsed times 5, 15, 30 and 60 min. The vertically averaged infection‐probability fields ${\left\langle P\right\rangle }_{z}$ in the reference case are shown in the upper row and the absolute differences $\Delta {\left\langle P\right\rangle }_{z}$ between the intervention and the reference case in percentage units are shown on the lower row. Infection probability is estimated from the LES‐predicted aerosol concentration data as described in Section 2.5. In this comparison, the near‐source area (horizontal distance <2 m, marked with a gray opaque disk) is not of primary interest since direct droplet transmission may also occur there (see Section 2.5).

Figure 8 shows how the air purifiers reduced the highest infection probabilities considerably depending on the location. The absolute reduction is significant within about 6 m of the source while the relative reduction is significant throughout the room. Comparison of the three‐dimensionally averaged infection‐probability values (see Section 2.5) to the reference case at 15, 30 and 60 min time instances yield −13%, −28% and −31% relative differences, respectively. It is observed that the relative differences remain roughly at a constant level after 30 min. After 5 min period the probability field outside the near‐source area remains low, which results in ill‐defined relative differences.

#### 3.2.2 Spatial partitioning with space dividers (DTC:DIV)

Aerosol particle concentrations in the DTC reference and with spatial partitioning with dividers are shown in Figure 5. The aerosol particle concentrations in the intervention and reference simulations remain similar throughout the simulations.

**FIGURE 9:**
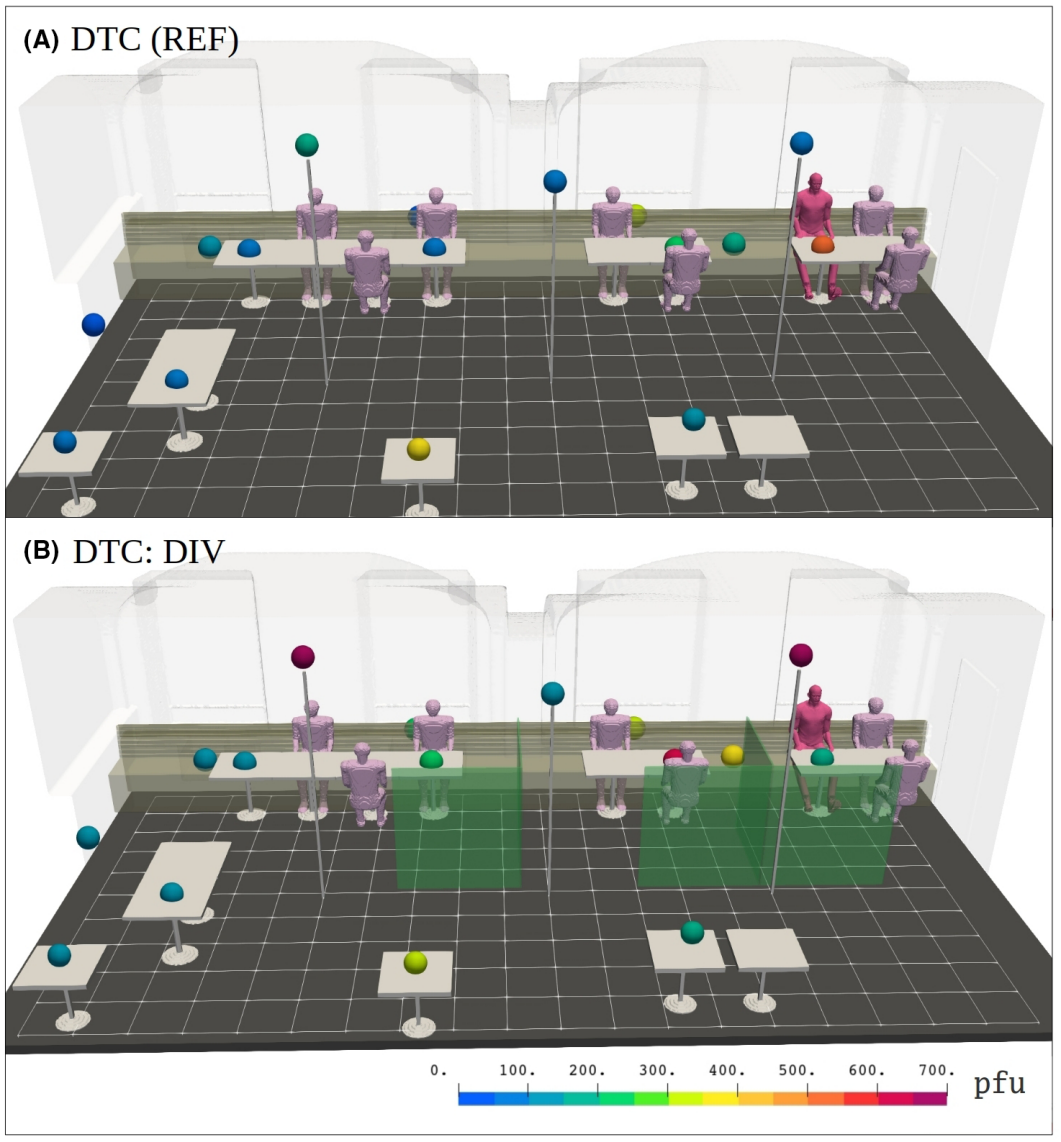
Identical visualization with Figure 7 but featuring (A) space‐divider table configuration (DTC) without the dividers and (B) with the dividers erected. No zero pfu counts were found.

In the deposition sample results, infectious viruses were found throughout the space, both in the reference and DTC:DIV intervention. The use of space dividers concentrated airborne viruses within the compartment close to the aerosol source as shown in Figure 9. Additionally, higher amounts were seen on deposition plates near the ceiling. Detailed information on deposition samples (pfu results for each plate) are shown in Table A5 in Appendix 2.

In the intervention with the space dividers, higher concentrations of infectious particles were captured by the Andersen impactor located close to the aerosol source, see Table 3. Similarly, the BioSpot sampler (See Table 3) captured close to a one order of magnitude higher virus concentration in the intervention compared with the reference. The locations of the Andersen impactors and BioSpot are shown in Figure 1. These results correlated with the LES model and deposition results.

**TABLE 3:** Simulations with space dividers DTC:DIV and the reference DTC (REF).

Andersen impactors	DTC (REF)		DTC:DIV	
Particle size *D*_p_ (μm)	A_near_	A_far_	A_near_	A_far_
7 ≤ *D*_p_	17	>700	700	234
4.7 ≤ *D*_p_ < 7	135	38	479	97
3.3 ≤ *D*_p_ < 4.7	481	166	>700	>700
2.1 ≤ *D*_p_ < 3.3	>700	>700	>700	>700
1.1 ≤ *D*_p_ < 2.1	>700	376	401	500
0.65 ≤ *D*_p_ < 1.1	353	363	430	376
BioSpot sampler	DTC (REF)		DTC:DIV	
	pfu/ml	pfu/L	pfu/ml	pfu/L
	4.00 × 10^3^	8.30	11.0 × 10^3^	22.90

*Note*: Infectious virus counts (pfu) in two different Andersen impactors and BioSpot sampler. See the caption of Table 2.

**FIGURE 10:**
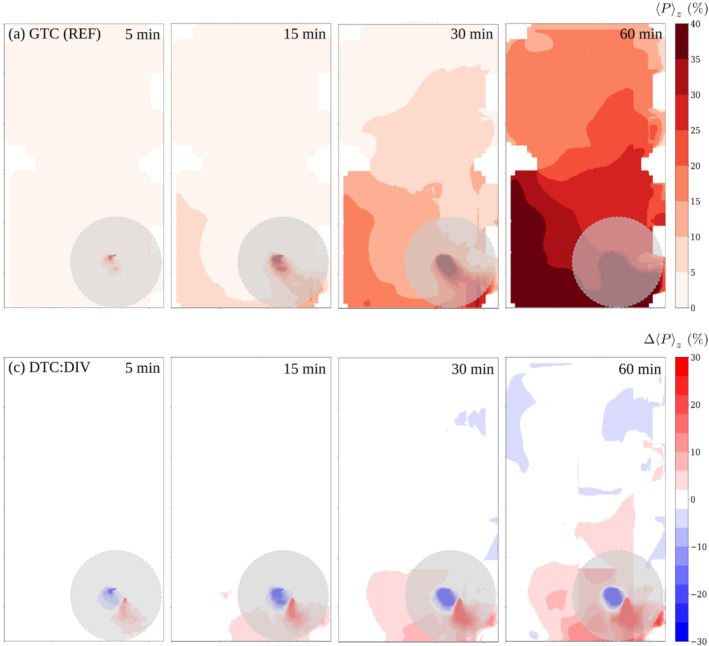
The evolution of infection probability with 100 qh^−1^ quanta rate for the GTC reference case (upper row), and the absolute difference in percentage units between the space‐divider intervention and GTC reference cases. The opaque gray disks having radius of 2 m indicate the near‐source zone within which direct droplet transmission may occur.

The estimated infection probability is shown in Figure 10 in the same fashion as for the GTC:FLT intervention. It must be noted here that for the LES results the GTC (REF) case is used as the reference since the DTC (REF) situation was not separately modeled by LES. The differences between the two reference setups were limited to table arrangement and 0.5 m difference in the nebulizer location. Figure 10 shows that infection probability in the compartment with the viral source increases and over time the probability rises also in the adjacent compartment. The effects of the space dividers outside the near‐source area are either negligible or even slightly adverse, which are reflected in the low spatially averaged infection‐probability differences 1%, −1% and 1% evaluated at 15, 30 and 60 min time instances, respectively.

All surface swab samples (A–N + Bunny chain, see Figure 1) remained negative regarding infectious viruses with space dividers. Finger tests from tables A and C as well as from the Bunny chain showed infectious viruses in both reference and intervention simulations. The finger test from couch N, near the source, was positive in the reference, and the test from the furthest sampling point, table D (8 m), remained negative in both reference and intervention simulations.

#### 3.2.3 Enhanced ventilation together with spatial partitioning (DTC:DIV + FLT)

**FIGURE 11:**
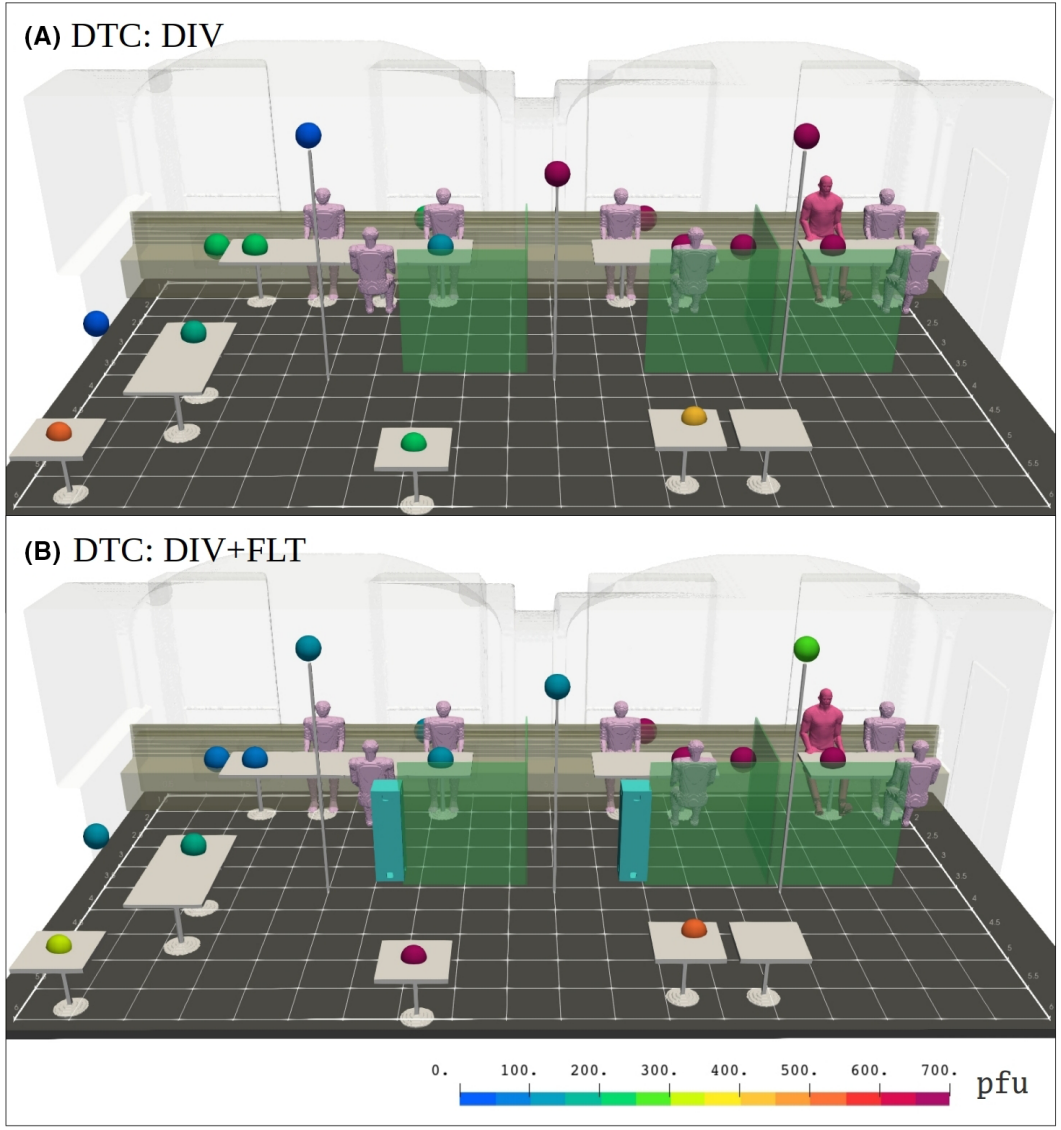
Identical visualization with Figure 7 but featuring space‐divider seating and table configuration (DTC) without the APs (A) and with the APs (B). It should be noted, that the reference (A) DTC:DIV without APs differs a bit from Figure 9B as the results are from different simulation. Again, no zero pfu counts are found.

**FIGURE 12:**
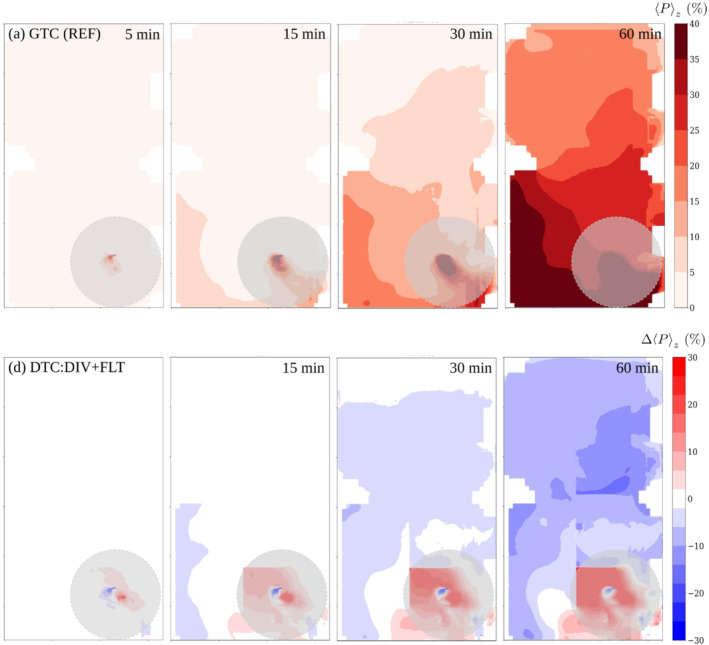
The evolution of infection probability with 100 qh^−1^ quanta rate for the reference case (upper row), and the absolute difference in percentage units between the combined space‐divider + air‐purifier intervention and reference cases. The opaque gray disks having radius of 2 m indicate the near‐source zone within which direct droplet transmission may occur.

The last studied risk‐reduction strategy combined the use of air purifiers and space dividers. The measured particle concentrations in the DTC reference and intervention DTC:DIV + FLT are presented in Figure 5. In this configuration, the deposition sample trend shows virus concentrations that are visibly diluted in the compartments situated farther from the infected person, but that remain elevated in the compartment with the source of infection. The deposition results from DTC:DIV + FLT are presented in Figure 11. For detailed information on deposition samples (pfu results for each plate) please see Appendix 2 for Table A6. In contrast to the LES results (See Figure 12) the pfu results also remained elevated in the compartment adjacent to the one containing the infected person.

Higher concentrations of infectious viruses were captured by the Andersen impactor placed near the aerosol source in the intervention compared to the reference simulations, possibly due to local peaks in the concentrations when using APs (see Table 4). The concentrations measured by the BioSpot were within a similar range as those of the reference (see Table 4) indicating that no major reduction in infectious viruses was detected from this distance when using space dividers and APs simultaneously. A similar trend was seen from the deposition results presented in Figure 11.

**TABLE 4:** Simulations with space dividers and air purifiers (DTC:DIV + FLT) and the reference (DTC:DIV (REF)).

Andersen impactors	DTC:DIV (REF)		DTC:DIV + FLT	
Particle size *D*_p_ (μm)	A_near_	A_far_	A_near_	A_far_
7 ≤ *D*_p_	54	9	>700	28
4.7 ≤ *D*_p_ < 7	143	7	>700	41
3.3 ≤ *D*_p_ < 4.7	118	139	>700	127
2.1 ≤ *D*_p_ < 3.3	>700	>700	>700	>700
1.1 ≤ *D*_p_ < 2.1	>700	>700	>700	387
0.65 ≤ *D*_p_ < 1.1	>700	413	417	353
BioSpot sampler	DTC:DIV (REF)		DTC:DIV + FLT	
	pfu/ml	pfu/L	pfu/ml	pfu/L
	5.0 ×10^3^	10.4	6.1 × 10^3^	12.7

*Note*: Viable virus counts (pfu) in two different Andersen impactors and BioSpot sampler. See the caption of Table 2.

The estimated infection probability using air purifiers and spatial partitioning are presented similarly as in the previous intervention cases in Figure 12 again using GTC (REF) as a reference instead of DTC (REF). The risk reduction was comparable to that seen in the GTC:FLT case (See Section 3.2.1) yielding −30%, −32% and −32% relative differences in spatially averaged infection probabilities at 15, 30 and 60 min, respectively. It should be recalled that these values are computed neglecting the near‐source where the infection risks have significantly risen due to the spatial partitioning.

Surface swab samples (A–N + Bunny chain, see Figure 1) were negative regarding infectious viruses. Finger tests from table A and couch N, near the source, showed infectious viruses in both reference and intervention simulations, while table C was positive in the intervention and Bunny chain on ceiling in the reference. Table D remained negative in both the reference and intervention.

## 4 DISCUSSION

Carefully planned multilayer virus transmission control strategies are needed to mitigate the pandemic.^42^ To this end, we examined the ability of airborne viruses to remain infectious during indoor dispersion and analyzed three different widely used risk‐reduction strategies:

1. augmenting filtration and ventilation capacity with air purifiers (GTC:FLT);
2. spatial partitioning with space dividers (DTC:DIV);
3. combination of both strategies (DTC:DIV + FLT).

The subsequent sections present the relevant discussion of the obtained results.

### 4.1 Aerosol generation, size range and dispersion

A nebulizer was used to simulate an infected person. The nebulization rate was chosen to be higher than the typical human emission rate of virus‐laden aerosol particles to ensure that the expected detection limit of the samplers was exceeded in all locations provided that the virus survived and dispersed throughout the space. This was justified since the results scale linearly with the nebulization rate, that is, they can always be rescaled to correspond to any chosen emission rate and are thus applicable to any variant when rescaled.

The median nebulized particle were 0.60–0.74 μm in diameter. These sizes are comparable to previous studies showing that nearly all of the particles generated by human respiratory activities are smaller than 5 μm^25^, ^43^, ^44^ and in line with Orton et al.^45^ showing unimodal particle emission at rest and during exercise 0.57–0.71 μm in diameter. Thus, our method for producing the aerosol particles was relevant for the respiratory context. Most of the respiratory pathogens, including also SARS‐CoV‐2, are found in particles smaller than 5 μm.^11^, ^46^, ^47^, ^48^ These small aerosol particles can remain airborne for a long time, accumulate indoors, travel with air currents as seen in our results, and can deposit in the lungs when inhaled.^49^, ^50^, ^51^ Furthermore, aerosol inoculations have been associated with more severe infection pathology even when caused by a lower viral dose compared to transmissions via other routes.^52^, ^53^

Enveloped viruses, such as influenza and SARS‐CoV‐2 have been observed mainly in aerosol particle sizes approximately 1–4 μm.^54^, ^55^ In our study, infectious viruses were most abundant in the size range of 1.1 μm < *D*_p_ < 4.7 μm and the size range 2.1 μm < *D*_p_ < 3.3 μm was showing >700 pfu in all simulations. This may indicate the most probable size range for catching the virus in this study; however, there are a lot of uncertainties in the generalization of this finding. This size range is larger than the measured median particle size of 0.60–0.74 μm, see Figure 6; however, it should be noted that we did not nebulize an uniform distribution of particles. This may have some indication that viral concentrations are either higher in a bit larger aerosol particles or more infectious viruses are lost due to drying in the smaller particles. On the other hand, we do not know how the viruses were distributed in different size particles and whether concentration occurs when the particles evaporate. It is also possible that virus infectivity is lost due to the mechanical stress the virions are exposed to on their way through the Andersen impactor, as the smallest size ranges are impacted in the last stages of the impactor. For future studies, it is important to include actual microbiological agents, such as virus particles or bacterial spores, rather than to draw conclusions purely from a non‐biological model aerosol to better understand these possible differences between viral and aerosol distributions. It should also be bear in mind that there is some variation regarding the culture Petri dishes as they are handmade. This variation may affect the Andersen impactor's stages if the Petri dish surface height changes.

We detected viable viruses throughout the space in every simulation. This proves that viruses must be transmitted in aerosol particles while maintaining infectivity in addition to larger droplets. This is in line with previous expectations that viable, airborne virus particles will spread as aerosol throughout a given indoor space, creating an infection risk despite long distances.^4^, ^21^, ^56^, ^57^, ^58^, ^59^, ^60^, ^61^ Clearly higher aerosol and virus concentrations were seen near the infection source. This was expected, and explains why physical distancing decreases infection risk as noted in a previous systematic review.^62^

### 4.2 Risk‐reduction strategies

Safety distances of at least 1–2 m between the measurement points were used in all configurations. As seen from the results, distance alone will reduce transmission risk for individuals further from the source, but still, the risk increases with increasing duration of exposure. Previous studies have detected airborne COVID‐19 transmission in restaurant spaces after 5–98 min exposure.^23^, ^63^, ^64^ However, in a study that measured the SARS‐CoV‐2 RNA in a canteen during the COVID wave in Italy in November 2020 air samples remained negative.^65^ This may follow from large air space, ventilation, and the possibility of non‐infected customers. Often a 15 min time frame is used as a limit value for contact tracing for people who have been closer than 2–4 m from the infected person. However, true “safe time” cannot be established. Duration of the exposure, ventilation conditions and the produced quanta rate strongly influence the overall inhaled dose of virus‐laden aerosol particles and the risk increases quickly with time when the quanta rate rises (e.g., highly transmittable variants such as delta or omicron, aerosol generating behavior such as loud speaking, shouting or singing^24^, ^25^, ^41^, ^66^).

We showed that APs, when used with or without space dividers augmenting the overall ventilation rate by 65%, reduced the average risk by 30%–32% compared to the reference case. This relative reduction was observed to manifest only after approximately 15 min of dispersion as APs can only reduce the aerosol concentration when the particles have reached the units. Open space with well‐mixed air results in more diluted virus concentrations throughout the space (See Figures 8 and 12). As often more than one cell infected with at least one viral particle is needed to cause an infection,^67^ the dilution lowers overall risk. Division of the space into compartments with APs further dilutes the concentrations in such compartments that do not contain infected individuals. Similar positive findings of APs have been showed in previous studies using aerosol measurements^68^, ^69^, ^70^, ^71^, ^72^, ^73^ and APs have been suggested as one measure to increase indoor safety during the pandemic.^74^, ^75^ In a comparison of two restaurant outbreaks the enhancement of indoor air dilution was associated with significantly lower secondary attack rate.^64^ Our results support these findings and advocate the use of APs in indoor environments where enhanced ventilation is needed. To achieve a desired level of risk‐reduction, it is important that the capacity of air purifiers is sized properly such that it augments the existing ventilation rate sufficiently. Similarly, it is important to choose filter material, such as HEPA filters, that will efficiently remove small aerosol particles. However, the ability to generate higher clean air delivery rates is more important than small differences in filtration ability (e.g., MERV13 vs. HEPA).^76^ Nevertheless, it is important to stress that augmented ventilation can never eliminate the risk of infection completely, but rather lower it. In this study, infectious viruses were always detected throughout the studied space.

Multiple guidance have proposed the use of physical barriers such as space dividers in prevention of SARS‐CoV‐2 transmission.^77^, ^78^ In multiple documents the partitioning is suggested to prevent flows of respiratory particles and thus reduce the probability of infection. Previous studies have suggested that barriers may reduce lateral spread by reducing mixing and blocking expiratory air jets.^57^, ^79^, ^80^ However, this is mainly true for ballistic droplets. The influence on dispersion of smaller particles is transient as seen in our results (See Video S1). At the same time aerosol particles can accumulate within the compartment, increasing the risk of infection for current and future occupants as the infective dose is reached faster.^81^

In our study, the aerosol particle concentrations in the DTC:DIV intervention and reference simulations remained overall similar throughout the simulations but increased within the source compartment.^10^ This is expected, as deposition of the small airborne aerosol particles onto the divider walls is deemed negligible as only a small fraction of the aerosol particles ends up in near contact with these surfaces.

Several researchers have suggested that short‐range airborne transmission is the most common route for respiratory infections in an indoor environment,^15^, ^16^, ^56^, ^82^, ^83^, ^84^, ^85^ and our results support this, even if risk‐reduction methods are applied. As seen, preventing transmission at close range without personal protective equipment is very challenging.

### 4.3 Turbulence and deposition

Traditional analytical models, such as the Wells‐Riley model^86^ and its extended form by Gammaitoni and Nucci,^39^ are based on the assumption of perfect and immediate mixing in the entire studied space. However, it is important to acknowledge the effects of the indoor turbulence and imperfect mixing on aerosol concentration and deposition. This has become possible through computational LES modeling employing current supercomputers.

The removal rate of aerosol particles from the space after nebulization was switched off at *t* = 60 min was estimated by using both the LES model and the extended Wells‐Riley model.^39^ When removal is modeled using LES, the half‐life is estimated to be 15 min in the reference case and 10 min with the air purifiers. The Wells‐Riley modeling was initiated from the LES‐predicted concentration at the nebulization cutoff, and it predicted the half‐lives to be as short as 9 min and 5 min, respectively. This is as expected since Auvinen et al.^27^ showed that the Wells‐Riley model strongly underestimates the concentration and thus infection probability due to the underlying assumption of spatially constant concentration. If the whole temporal evolution of the concentration shown in Figure 3 were modeled using the Wells‐Riley model, it would predict not only an excessively rapid removal processes but also nearly four times smaller concentration values during the aerosolization period 0 min ≤ *t* ≤ 60 min than the LES model. Our results complement the previous understanding of the limitations of the analytical Wells‐Riley model, see for example,^87^ and provide a more comprehensive approach.

Indoor turbulence affects also particle deposition. Although the method to detect infectious viral particles deposited onto host culture plates is accurate and much used as such, the deposition onto the plates themselves involves high uncertainties and unknown spatial variability. It is important to understand that the pfu results represent 1 h cumulative aerosol particle depositions on a plate under turbulent flow conditions. The deposition result should be treated primarily as positive and negative, and only indicative regarding the quantity.^88^ This is because the deposition‐plate results depend not only on local virus‐aerosol concentration but also on the flow field, its mean and turbulent fluctuating parts and on the local aerosol size distribution. However, positive pfu results do show the presence of infectious viruses in aerosol particles, which have remained airborne for a prolonged period. Both mean downward flow and strong turbulence (intense vertical velocity fluctuations) enhance the flux toward a plate and thus tend to increase deposition onto it. This is the assumed reason for the somewhat counter‐intuitive increase in pfu results on the deposition plates near APs in the GTC:FLT and DTC:DIV + FLT interventions (See Figures 7 and 11).

Discussion of the role played by deposition to surfaces during COVID‐19 pandemic has been intense. In April 2021 the United States Centers for Disease Control stated that the risk of fomite transmission was low, generally less than 1:10 000.^89^ In a previous restaurant study of a COVID‐19 outbreak no evidence of fomite transmission was obtained.^90^ We detected infectious viruses from all deposition and air samples, but interestingly, we were not able to detect infectious viruses next to the deposition sample locations by using traditional surface sampling method. Only finger surface samples showed positive results. Although these results are primarily indicative, they do demonstrate that swab tests may fail to detect infectious viruses, although widely used to detect viral RNA on inanimate surfaces.^11^, ^91^ One factor supporting the use of sampling methods other than swabbing is that these techniques may more easily obtain samples from a larger area, which has been associated with a higher number of positive surface results.^12^ Overall this finding raises multiple questions: (1) Are current surface sampling methods sensitive enough and do they need more optimization? (2) Is the loss of infectivity on surfaces a true finding, as in laboratory conditions surface sampling methods seem to be able to detect viable viruses and we know from deposition samples that there have been infectious viruses at the sampled area? (3) Is the loss of infectivity due to drying on a surface or other environmental reasons?

### 4.4 Limitations

Our experimental study has been a learning process to us and have several limitations that can be further addressed in the future studies. In this study we decided to use 60 min simulation time. Without the purifiers the increase continued and long‐time equilibrium was estimated to be reached approximately at 80 min. The air purifiers enhanced mixing and thus shortened the mixing time when compared to the case without the purifiers. Therefore, with the purifiers on *c* ^+^ reached the long‐time equilibrium state already at about *t* = 35 min. However, we decided to use 60 min simulation times for all intervention cases since clear conclusions can be drawn even without reaching the final asymptote.

The aerosol number concentration time series, and especially the APS_near_, show that the nebulization rate was not constant over the 90 min period (See Figure 4). This is most likely because particle production rate is sensitive to salt and virus concentrations and the nebulized liquid was in three separately mixed bottles, which were used to refill the nebulizer. However, in this single experiment, it did not have an effect on the main findings. Later, when comparing the intervention simulations to their references (60 min simulations), the liquid bottles were filled from the same, larger quantity of liquid to ensure the same concentration in each bottle to avoid changes in particle production.

In the DTC:DIV + FLT reference APS detected lower concentrations than expected. It is seen that the concentration remains low especially after liquid for the nebulization is changed in the middle of the simulation. This variability during the nebulization may be due to temperature changes as the liquid concentration was constant and liquid was originally from the same bottle. The result indicates that nebulization is a rather sensitive process to any changes.

The detection range of APS 0.5–20 μm covers particle size distribution nebulized in the air. Although small aerosol particles <0.5 μm can exist, their ability to carry pathogens is restricted to the point when the size of the pathogen exceeds the size of the particle. Further studies are needed to understand the relative importance of the <0.5 μm aerosol particles. Fine droplets from 20 μm to approximately 100 μm, although capable of remaining suspended in the air for seconds, mainly fall in the close region from the source. We excluded that area from analysis as also direct droplet transmission can theoretically occur within distances less than roughly 2 m.

In the future studies, it remains a challenge to further develop methods that have a higher sensitivity, lower infectivity losses and an ability to quantify infectious viruses to further analyze viral and modeling results.

### 4.5 Summary and conclusions

This study combined Phi6 surrogate virus experiments and high‐resolution large‐eddy simulation modeling to address (1) the viral transmission mechanisms in indoor space, (2) infectivity of the virus in air and on surfaces, (3) indoor aerosol dispersion, and (4) the effects of risk‐reduction strategies on infection probability. The exploitation of LES jointly with the experimental results enables a more informative interpretation of the measurements, facilitating a more complete risk assessment. We showed that Phi6 virus was dispersed in aerosol particles throughout the studied restaurant space while maintaining infectivity. The results from infectious virus measurements and LES‐modeling showed similar trends for infection probability in all simulations. Augmenting the ventilation capacity with air purifiers, with or without space dividers, reduced the infection‐probability levels within the room, but the relative reduction manifested with a lag due to the gradual nature of aerosol dispersion. The overall net effect of space dividers was observed negligible. Thus, the use of space dividers alone is not considered a strategy to mitigate the potential for infection, and could instead even increase the risk locally. The indoor flow field significantly affects particle concentrations, often giving rise to highly variable infection‐probability distributions. We believe that this study is a step closer to understanding viral transmission in indoor environments outside the laboratory and thus brings valuable information to the fight against COVID‐19 and other respiratory infections.

## AUTHOR CONTRIBUTIONS

L‐M.O., M.A, J.K, M.R, A‐P.H., S.L., E.S., T.G., A.H., and N.A involved in conceptualization. L‐M.O., M.A, J.K, R.M., S.L., S.S., H.V., T.G., A.H., and N.A. involved in data curing. L‐M.O., M.A, J.K, M.R., T.G., A.H., and N.A. involved in formal analysis. L‐M.O, A‐P.H., S.L., L.M., E.S., A.G., T.S., A.H, and N.A. acquired the funding. L‐M.O., M.A., J.K., R.M., L.M., S.S., J.S., H.V., T.G., A.H., and N.A. involved in the investigation process. L‐M.O., M.A., J.K., S.L., L.M., E.S., S.S., T.G., A.H., and N.A. involved in the methodology. L‐M.O, A‐P.H., E.S., A.G., T.S., and N.A. administered the project. L‐M.O., M.A, J.K, M.R, S.L., E.S., A.G., T.G., A.H., and N.A. provided the resources. J.K., M.A., A.H., and T.G. provided the software. L‐M.O., M.R., A‐P.H., E.S., A.G., A.H., and N.A. supervised the manuscript. J.K. and T.G. involved in validation. M.A. and J.K. involved in visualization. L‐M.O., M.A., R.M., T.G., A.H., and N.A. wrote the original draft. All authors reviewed and edited the article.

## CONFLICT OF INTEREST

The authors declare no conflicts of interest.

## Supporting information


Video S1.

## Figures and Tables

**FIGURE A1:**
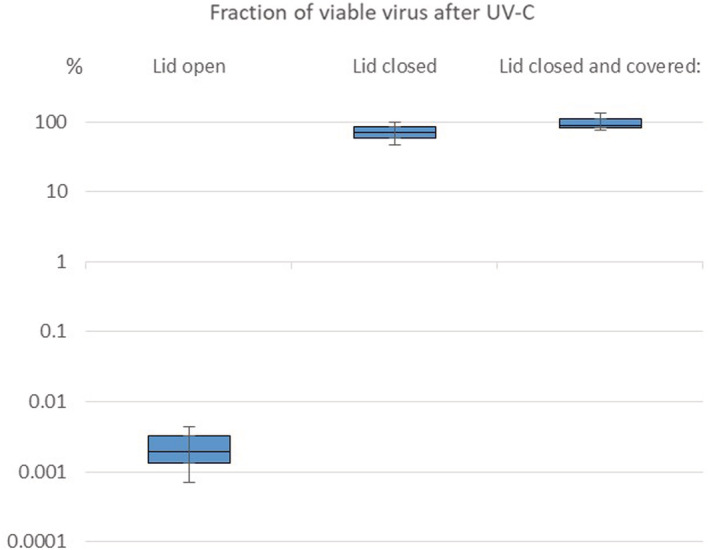
Effect of the UV‐treatment on viable virus fraction.

## Data Availability

The data supporting the virus infectivity and conclusions of this study are available within the article and in Appendix 2. More extensive background data is available from the corresponding author upon reasonable request. The PALM model system is freely available at http://palm‐model.org and distributed under the GNU General Public License v3 (http://www.gnu.org/copyleft/gpl.html). However, the simulations presented in this article were performed using a modified code based on PALM revision 4786. This modified source code (4786M) is available at https://doi.org/10.5281/zenodo.5596111.^92^

## APPENDIX 1

### INACTIVATION OF VIRUS BY UV‐C IRRADIATION

The effective irradiance (*E*_eff_) of the UV disinfection lamp Svetolit UV‐C 600 (253.7 nm, 600 W, LIT Uv Elektro GmbH) was measured during disinfection of airborne viral aerosol particles after nebulization. The measurements were carried out by Solar Light's Radiometer (PMA2100) and UV Radiation Safety Sensor (PMA 2120) after 5 min switching the UV lamp on. The measurement height was 1 m and distances from the lamp were 1, 2 and 4 m. The *E*_eff_ is spectrally weighted irradiance within the UV wavelength range from 180 nm to 400 nm. The unweighted irradiance of 253.7 nm wavelength radiation was obtained by multiplying the *E*_eff_ by a factor of 3. Virus inactivation by UV was studied by exposing three petri dishes, each with three 20 μl droplets of the nebulization solution to UV for 10 min + 10 min. Plate 1 was left lid open, plate 2 was covered with a see‐through plastic lid and plate 3 was covered with plastic lid and a dark metal cover. Virus titers from each droplet were measured by plaque assay. The results are shown in Figure A1.

## APPENDIX 2

### SAMPLING POINT INFORMATION AND ADDITIONAL RESULTS

**TABLE A1:** Sampling point information

Sampling point	Sampling location	Distance from nebulizer (m)
A	Table	0
B	Table	3.0
C	Table	5.5
D	Table	8.0
E	Table	8.5
F	Table	8.8
G	Table	4.5
H	Table	6.5
I	Table	9.2
J	Back wall	10.0
K	Windowsill	5.5
L	Windowsill	3.0
M	Couch	8.5
N	Couch	0.8
O	Roof sculpture “Bunny chain” head	1.3
P	Roof sculpture “Bunny chain” mid	4.5
Q	Roof sculpture “Bunny chain” tail	7.0

**TABLE A2:** Virus concentration (pfu/ml) of the nebulizer solution.

Simulation	Batch (150 ml)	Start concentration (pfu/ml)	End concentration (pfu/ml)
GTC:FLT		1.90 × 10^10^	
GTC:FLT	1		1.10 × 10^10^
GTC:FLT	2		6.30 × 10^9^
GTC:FLT REF	3		1.50 × 10^10^
GTC:FLT REF	4		1.40 × 10^9^
DTC:DIV		1.36 × 10^10^	
DTC:DIV	1		1.10 × 10^10^
DTC:DIV	2		1.80 × 10^9^
DTC:DIV REF	3		2.50 × 10^9^
DTC:DIV REF	4		4.50 × 10^9^
DTC:DIV + FLT		1.50 × 10^10^	
DTC:DIV + FLT	1		2.40 × 10^9^
DTC:DIV + FLT	2		2.20 × 10^9^
DTC:DIV + FLT REF	3		7.90 × 10^9^
DTC:DIV + FLT REF	4		3.70 × 10^9^

Abbreviations: DIV, dividers; DTC, divider table configuration; FLT, filtration; GLC, generic table configuration.

**TABLE A3:** Virus deposition rate (pfu/30 min) control during the first and second half of nebulizing time.

Simulation	Nebulizing time (min)	Virus deposition (pfu)
GTC:FLT	1st 30	270
GTC:FLT	2nd 30	140
GTC:FLT REF	1st 30	>700
GTC:FLT REF	2nd 30	>700
DTC:DIV	1st 30	>700
DTC:DIV	2nd 30	130
DTC:DIV REF	1st 30	127
DTC:DIV REF	2nd 30	199
DTC:DIV + FLT	1st 30	268
DTC:DIV + FLT	2nd 30	253
DTC:DIV + FLT REF	1st 30	>700
DTC:DIV + FLT REF	2nd 30	>700

Abbreviations: DIV, dividers; DTC, divider table configuration; FLT, filtration; GLC, generic table configuration.

**TABLE A4:** Infective virus numbers on deposition plates.

Simulation	Sampling point	Intervention infective viruses (pfu)	Reference infective viruses (pfu)
FLT	A	TMC	TMC
FLT	B	706	199
FLT	C	487	564
FLT	D	423	332
FLT	E	178	207
FLT	F	316	127
FLT	G	285	171
FLT	H	309	183
FLT	I	265	251
FLT	J	137	133
FLT	K	152	177
FLT	L	444	TMC
FLT	M	159	302
FLT	N	264	TMC

Abbreviations: FLT, filtration; TMC, too many to count.

**TABLE A5:** Infective virus numbers on deposition plates.

Simulation	Sampling point	Intervention infective viruses (pfu)	Reference infective viruses (pfu)
DIV	A	142	TMC
DIV	B	386	234
DIV	C	1	54
DIV	D	TMC	61
DIV	E	TMC	41
DIV	F	N/A	N/A
DIV	G	436	71
DIV	H	95	N/A
DIV	I	119	94
DIV	J	435	20
DIV	K	80	53
DIV	L	86	173
DIV	M	211	N/A
DIV	N	127	57
DIV	Bunny chain head	101	183
DIV	Bunny chain mid	112	85
DIV	Bunny chain tail	N/A	69

Abbreviation: DIV, dividers; TMC, too many to count.

**TABLE A6:** Infective virus numbers on deposition plates.

Simulation	Sampling point	Intervention infective viruses (pfu)	Reference infective viruses (pfu)
DIV + FLT	A	TMC	TMC
DIV + FLT	B	TMC	TMC
DIV + FLT	C	158	66
DIV + FLT	D	85	174
DIV + FLT	E	105	154
DIV + FLT	F	N/A	N/A
DIV + FLT	G	320	183
DIV + FLT	H	174	TMC
DIV + FLT	I	TMC	TMC
DIV + FLT	J	44	130
DIV + FLT	K	126	N/A
DIV + FLT	L	N/A	N/A
DIV + FLT	M	N/A	120
DIV + FLT	N	TMC	TMC
DIV + FLT	Bunny chain head	254	TMC
DIV + FLT	Bunny chain mid	103	TMC
DIV + FLT	Bunny chain tail	138	49

Abbreviation: DIV, dividers; FLT, filtration; TMC, too many to count.
